# Functional Characterization and Toxicological Study of Proanthocyanidins in Weaned Pigs

**DOI:** 10.3390/toxins15090558

**Published:** 2023-09-07

**Authors:** Jiahao Liu, Yong Qiao, Bing Yu, Yuheng Luo, Zhiqing Huang, Xiangbing Mao, Jie Yu, Ping Zheng, Hui Yan, Yan Li, Jun He

**Affiliations:** 1 Institute of Animal Nutrition, Sichuan Agricultural University, Chengdu 610030, China; liujiahao@stu.sicau.edu.cn (J.L.); qiaoyong@newhope.cn (Y.Q.); ybingtian@163.com (B.Y.); luoluo212@126.com (Y.L.); zqhuang@sicau.edu.cn (Z.H.); acatmxb2003@163.com (X.M.); jerryyujie@163.com (J.Y.); zpind05@163.com (P.Z.); yan.hui@sicau.edu.cn (H.Y.); m18782044216@163.com (Y.L.); 2 Key Laboratory for Animal Disease-Resistance Nutrition of China Ministry of Education, Chengdu 610030, China; 3 Feng Lan Sci-Tech Co., Ltd., Chengdu 610097, China

**Keywords:** proanthocyanidins, safety, growth performance, weaned pigs, intestinal epithelium

## Abstract

Proanthocyanidin (PRO) has been implicated in a variety of biological functions, such as antibacterial, antioxidant, and anti-obesity effects. However, little is known about its safety dose for animals. To explore its safety and effect on growth performance and intestinal health, thirty weaned pigs were divided into five groups and fed with basal diet containing 0, 30, 300, 600, and 1200 mg/kg PRO for 42 days. Results showed that PRO supplementation at 30 and 300 mg/kg significantly decreased the feed/gain ratio (F:G) and diarrhea rate (*p* < 0.05). PRO also increased the digestibilities of dry matter, ether extract, gross energy, and ash (*p* < 0.05). Interestingly, PRO not only elevated the villus height and the ratio of villus height to crypt depth (V/C) in duodenum and jejunum (*p* < 0.01), but also decreased the crypt depth in the duodenum (*p* < 0.01). Moreover, PRO supplementation at 30, 300, and 600 mg/kg elevated the expression levels of mucin 1 (*MUC1*), *MUC2*, and fatty acid transport protein 1 *(FATP-1*) in the duodenum (*p* < 0.05). The expression levels of *FATP-4* in jejunum and ileum were also elevated by PRO (*p* < 0.05). Importantly, histopathological findings of tissues (e.g., heart, liver, kidney, spleen, lungs, pancreas, thymus, mesenteric lymph nodes, stomach, small intestine), serum clinical chemistry, and major hematological parameters were not affected by PRO supplementation. These results suggest that PRO may act as a safe and effective supplement to decrease F:G and improve intestinal health in weaned pigs.

## 1. Introduction

PRO are oligo- or polymers of monomeric flavan-3-ols produced as an end-product of the flavonoid biosynthetic pathway, which are widely distributed in flowers, nuts, fruits, bark, and seeds of various plants [1,2]. The PRO degree of polymerization can range between 3 and 11 [1]. The polymerization of flavan-3-ols in PRO are linked by C-C and C-O-C bonds [3,4]. Grape seeds are one of the most abundant sources of PRO [5]. Antioxidant activity is the primary biological characteristic of PRO, which is 50 times that of vitamin E and 20 times that of vitamin C [6]. Besides antioxidant capacity, PRO also has been proven to possess extensive beneficial health properties, such as antibacterial, anti-inflammatory, anti-diabetic, and anti-obesity in animals and human studies [7,8,9]. For example, diet supplementation with PRO attenuated trinitro-benzene-sulfonic acid-induced recurrent ulcerative colitis in rats by downregulating the expression levels of tumor necrosis factor-alpha, Phosphorylated inhibitor kappa B kinase, and the translocation of nuclear factor kappa-B [10]. Studies showed that PRO ameliorated peripheral insulin resistance and disorders of lipid metabolism in vivo via inhibiting adipogenesis and improving mitochondrial function [11,12]. Moreover, PRO has also been implicated in regulating the growth of intestinal epithelial mucosa by up-regulating the tight-junction protein (TJP) expression levels (e.g., ZO-1, occludin) [13,14].

Herbs and spices have been used for medicinal purposes since the earliest civilizations to prevent or cure many diseases, and a quarter of the world’s medicines are prepared from plants [15,16]. However, research reports showed phytochemicals or plant extracts are toxic to animals above a certain dose, such as having carcinogenic, nephrotoxic, hepatotoxic, and gastrointestinal effects [17]. The PRO dose in previous studies was very low (250 mg/kg or 400 mg/kg feed), and the impact of high-dose PRO on animals’ health is still unclear, especially in young pigs [18,19]. Therefore, it is pretty essential to evaluate the safety of plant extracts PRO. Moreover, previous studies mainly focused on the antioxidant and anti-inflammatory abilities of PRO, with limited research on its functional characteristics, such as intestinal barrier function and digestive absorption capacity in pigs. Therefore, this study uses weaned piglets as the research model to explore the functional characterization and safety evaluation of PRO.

## 2. Results

### 2.1. Growth Performance and Diarrhea Rate

The results of dietary PRO supplementation on growth performance and diarrhea rate are shown in Table 1. The F:G in 30 and 300 mg/kg PRO-supplemented groups were greater than those in the CON group (*p* < 0.05); there was an improving trend in the 600 PRO-supplemented groups (0.05 < *p* < 0.10). Meanwhile, Supplementation of 30, 300, and 1200 mg/kg PRO led to a decrease in the diarrhea rate (*p* < 0.05). There is no difference in ADFI, FBW, and ADG between the PRO groups and the CON group.

### 2.2. Nutrient Digestibility

The results of dietary PRO supplementation on apparent total tract digestibility of nutrients are presented in Table 2. PRO supplementation from 0 to 1200 mg/kg increased the apparent digestibility of EE, GE, DM, and ASH contrast with the CON group (*p* < 0.05). However, there was no difference in CP with increasing levels of PRO.

### 2.3. Intestinal Morphology

**Figure 1:**
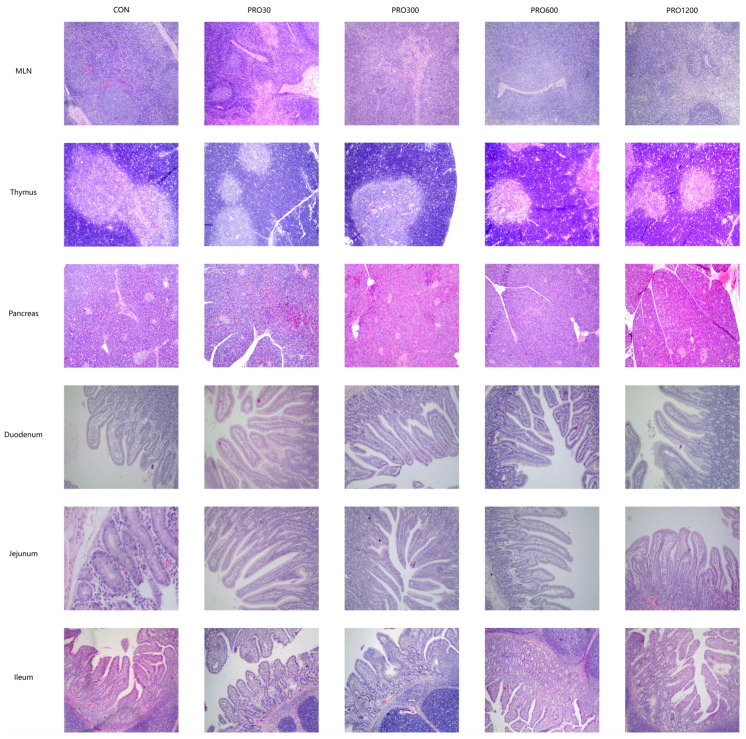
Effects of PRO on histopathological findings of pancreas, thymus, mesenteric lymph nodes, stomach, duodenum, jejunum, and ileum in weaned pigs (H&E; ×100). Con, pigs were fed a basal diet; PRO30, PRO300, PRO600, PRO1200, pigs were fed a basal diet supplemented with 30, 300, 600, or 1200 mg/kg PRO. MLN, mesenteric lymph nodes.

The results of dietary PRO supplementation on intestinal morphology are presented in Table 3 and Figure 1. Compared with the CON group, the PRO-supplemented increased the VH and V/C in duodenum and jejunum (*p* < 0.01). Moreover, PRO reduced the CD in jejunum.

### 2.4. Intestinal Barrier and Nutrition Transport Gene Expression Level

**Figure 2:**
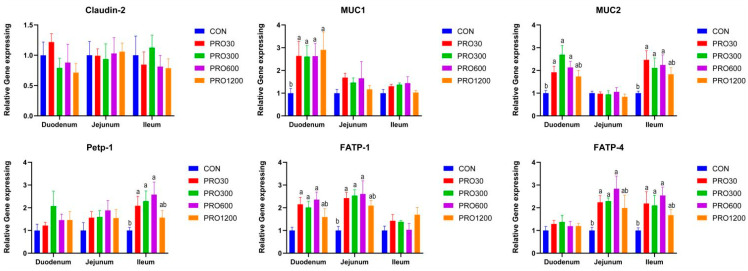
Effects of PRO on intestinal barrier and nutrition transport gene expression level. Con, pigs were fed a basal diet; PRO30, PRO300, PRO600, PRO1200, pigs were fed a basal diet supplemented with 30, 300, 600 or 1200 mg/kg PRO; a, b mean values within a row with unlike superscript letters were significantly different (*p* < 0.05).

The results of dietary PRO supplementation on gene expression levels related to intestinal epithelial functions are presented in Figure 2. Compared with the CON group, pigs receiving 30, 300, and 600 mg/kg PRO diet had higher expression levels of *MUC1* in duodenum and *MUC2* in duodenal and ileum (*p* < 0.05). Moreover, the 30, 300, and 600 mg/kg PRO diet-supplemented group improved the expression levels of *FATP-1* in duodenum and *FATP-4* in jejunum and ileum (*p* < 0.05). The ileal *Petp-1* expression level was also elevated upon 30, 300, and 600 mg/kg PRO feeding (*p* < 0.05).

### 2.5. Clinical Chemistry Parameters

The results of dietary PRO supplementation on clinical chemistry parameters are presented in Table 4. Most serum parameters show no differences between PRO supplemented and CON groups. On days 22 and 43, supplementation of 30 mg/kg PRO decreased the serum TC concentration (*p* < 0.05) and tended to increase serum ALT concentration compared with the CON group (0.05 < *p* < 0.01).

### 2.6. Hematological Parameters

The results of dietary PRO supplementation on hematological parameters are presented in Table 5. The hemoglobin concentration and hematocrit value were higher in dietary supplemented with 30 mg/kg PRO group than in the CON group at the beginning of the experiment (*p* < 0.05). Over the full study period, there were no differences between the CON and the four other groups in any other hematological parameters, such as WBC, RBC, HGB, PLT, lymphocytes, and neutrophils.

### 2.7. Relative Weights of Organ

The results of dietary PRO supplementation on relative weights of organs are presented in Table 6. There were no biologically meaningful differences in the relative weights of organs among all groups during the study, including liver, kidney, spleen, heart, and lungs.

### 2.8. Histopathological Findings

**Figure 3:**
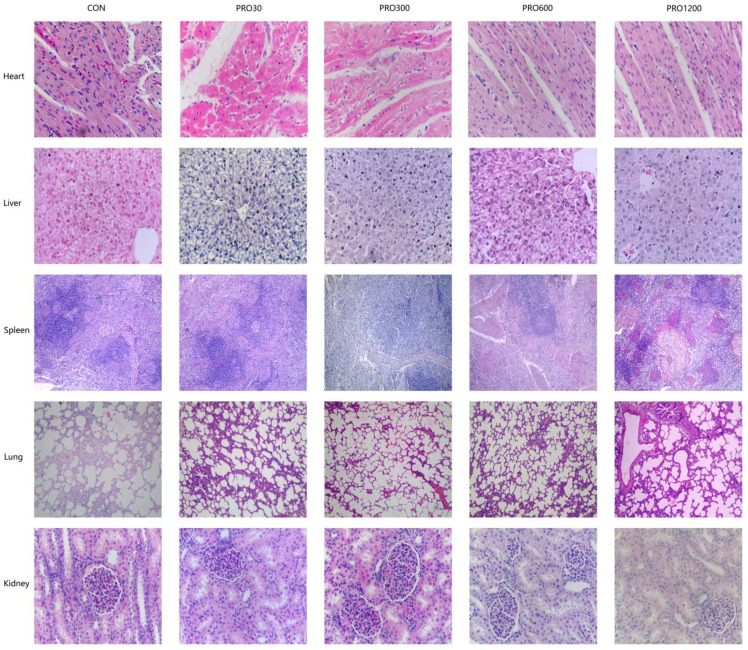
Effects of PRO on histopathological findings of heart, liver, kidney, spleen, and lungs in weaned pigs (H&E; ×100). Con, pigs were fed a basal diet; PRO30, PRO300, PRO600, PRO1200, pigs were fed a basal diet supplemented with 30, 300, 600, or 1200 mg/kg PRO.

The results of dietary PRO supplementation on Histopathological findings are presented in Figure 1 and Figure 3. Our histological examination of the heart, liver, kidney, spleen, lungs, pancreas, thymus, mesenteric lymph nodes, stomach, duodenum, jejunum, ileum, and cecum did not reveal any pathological changes. The detail analyses are as follows: The structure of the mucosal layer and the arrangement of the intestinal villi are normal; the structure of liver lobules is clear, the liver cords are arranged in an orderly manner, the central venous blood content of the cell morphology is normal; the pancreatic lobes, and the morphology of pancreatic islets A, B cells and adipocytes were normal; normal distribution of white pulp, red pulp, and lymphocytes; the epithelial cells of the renal tubules are arranged neatly, and the glomerulus and renal capsule are normal; the morphology, structure, and size of the alveoli and cardiomyocytes are normal; no obvious abnormalities in lymph tissue and lymph sinuses.

## 3. Discussion

In the present study, the maximum dose of PRO added to the feed of weaned piglets was 1200 mg/kg, which is approximately 48 times the optimal dose level (25 mg/kg) in some recent animal nutrition studies [20,21]. Numerous studies have indicated that dietary PRO plays a crucial part in beneficial effects for health issues including a powerful antioxidant capacity and possessing immunomodulatory activity in humans and animals. For example, a study in broiler chicks showed that PRO can significantly improve their ADG and antioxidant capacity [22]. Moreover, PRO as a type of phenolic compound is capable of reducing the colonization of a broad spectrum of Gram-negative and Gram-positive bacteria on the intestinal mucosa at a dose of 60 mg/L or 30 mg/L, which may alleviate the inflammation and injury of the intestinal barrier [23,24]. In this study, we observed that the growth performance was improved in 30 or 300 mg/kg PRO-supplemented piglets, as indicated by a decrease in F:G and diarrhea rate. The results were consistent with a previous study that dietary supplementation with 250 mg/kg PRO decreased the diarrhea rate and improved growth performance by reducing intestinal permeability by a urinary lactulose to manmitol ratio test in weaning rats [18]. Moreover, some similar research also reported that feeding polyphenol-rich plant extracts from grape or hop could decrease the F:G in growing pigs [25].

Weaning is a critical developmental window in a neonatal porcine. At this stage, Changes in feeding patterns and withdrawal of maternal passive immune protection may increase the susceptibility of piglets to pathogens such as *Escherichia coli*, which could result in inflammation and damage to the intestinal epithelium [26,27]. The integrity of the intestinal villus-crypt structure has been considered as a symbolic indicator to evaluate the intestinal epithelium function, such as intestinal barrier integrity, nutrient digestion, and absorption capacity of the small intestine [28,29]. In our present study, dietary PRO supplementation not only exerted a positive effect on intestinal morphology, which resulted in an increase in duodenal and jejunal VH, but also increased the digestibilities of DM, GE, EE, and ASH contrast with the CON group. The improvement in growth performance may be attributed to increased nutrient digestibility and improved intestinal morphology [30]. Similarly, previous studies indicated that dietary polyphenol-rich grape products were beneficial in increasing the small intestinal VH: CD in chicks and piglets [31,32,33]. Moreover, a study on rats also showed that supplementation with PRO could improve the intestinal barrier function the same as ZnO by improving the expression of intestinal mucosal tight junction proteins [18]. Based on those findings, our studies suggested that dietary PRO supplementation may alleviate intestinal damage caused by weaning stress by improving intestinal morphology.

The intestinal epithelial barrier is composed of the mucus layer, intestinal epithelial cells, and their tight junctions [34]. Under normal conditions, the intestinal tract is protected by a mucus layer with secreted (e.g., *MUC2*, *MUC5B*, and *MUC19*) and cell-surface mucins (e.g., *MUC1*, *MUC12*, and *MUC20*) from pathogenic microbes and toxin-induced damage [35]. Once the mucus layer in the gut is damaged, the intestinal epithelium surface will be exposed to a multitude of noxious numerous macromolecules and microorganisms, which may lead to a continuous immune reaction. We found that PRO significantly improved the expression level of *MUC1* in duodenum and *MUC2* in duodenum and ileum, which was similar to a previous report using a rat model [13,14]. *MUC2* plays a critical role in intestinal epithelial protection, the highly glycosylated cysteine residues located in the N and C termini of *MUC2* enhance the hydrophilicity of mucins and are beneficial for the lubrication of the intestinal mucosa [36]. Moreover, commensal bacteria can occupy microbial binding sites by binding to the intestinal mucosa through oligosaccharide linkage structures on *MUC2*, which protects against pathogen invasion [37]. Interestingly, dietary supplementation of PRO improved the expression levels of *FATP-1* in duodenum and *FATP-4* in jejunal and ileum. The ileal *Petp-1* expression level was also elevated upon PRO feeding. *FATP-1* and *FATP-4* are major members of the fatty acid transporter family that are involved in the cellular utilization and storage of long chain fatty acids and the regulation of the level of fatty acyl-Coenzyme A’s [38,39]. PepT1 is responsible for nutrient-derived di- and tripeptide transport [40]. These findings suggested that dietary PRO is beneficial in improving intestinal epithelial functions for weaned pigs, which may contribute to growth performance.

Those results of serum clinical chemistry and hematological parameters were similar to those of previous studies in weaned pigs, and hardly any differences were found between the PRO groups and the CON group at all stages [41,42,43]. Serum ALT was increased in pigs fed 30 and 300 mg/kg PRO compared with that in 600 and 1200 mg/kg PRO in the later period. For ALT, a biomarker of hepatic inflammation, every treatment value was within previous limits observed in weaned pigs [44,45,46], indicating that the numerical difference between groups is not biologically meaningful. Hemoglobin concentration, whose clinical significance is essentially the same as that of hematocrit value, was inversely associated with lung disease [47]. In this study, the hemoglobin concentration and Hematocrit value were higher in the 30 mg/kg PRO supplementation group than in the CON group, but this difference disappeared after the start of the experiment, and all concentrations were within the normal biological range for the weaned pigs [48]. Therefore, those findings were not considered to be related to the diets. Interestingly, with the 30 mg/kg PRO exposure, the serum TC concentration of weaned pigs showed a reduction trend. A study in rats demonstrated that supplemented 25 mg/kg PRO could decrease VLDL concentration through interference with genes involved in VLDL assembly (e.g., Microsomal Triglyceride Transfer Protein and diacylglycerol O-acyltransferase 2) [49]. Moreover, other studies also showed that PRO ameliorated peripheral insulin resistance and disorders of lipid metabolism in vivo via inhibiting adipogenesis and improving mitochondrial function [11,12]. These results suggest that PRO supplementation is beneficial to improve lipid metabolism in animals.

The internal body organ weight is a crucial parameter in assessing the safety of drugs [50,51]. Our results found that the PRO supplementation from 0 mg/kg to 1200 mg/kg in diets did not affect the relative weights of organs (e.g., heart, lungs, spleen, liver, and kidney). Moreover, histopathological examination of various internal vital organ sections of all pigs were also shown to be normal and showed no evidence of gross abnormalities. Therefore, it could be concluded that the addition of 48 times the optimal dose level of PRO to pig feed is safe and produces no adverse effects on histological, clinical chemistry, or hematological indicators in pigs.

## 4. Conclusions

PRO supplementation decrease F:G and improve intestinal health in weaned pigs, which are contributed by improved intestinal epithelial barrier function, apparent total tract digestibility of nutrients, as well as improved nutrition transport genes expression level. PRO has no toxic effects at a dose below 1200 mg/kg, as indicated by normal serum clinical chemistry, hematological parameters, and organs. These results suggest that the PRO may act as a potential and safety supplement for the weaned pigs breeding.

## 5. Materials and Methods

The animal experiment in this study was approved by the Animal Care and Use Committee of Sichuan Agricultural University (Chengdu, China, No. 20220716). PRO (Cat no. DAT00140, with a purity of PRO ≥ 95%, of which Oligomeric PRO ≥ 85%, catechine ≥ 1.52%, epicatechine ≥ 2.41%) was purchased from Guilin Feng peng Biological Technology Co., Ltd. (Guangxi, China).

### 5.1. Experimental Design, Diet, and Animal Housing

Thirty male weaned pigs (Duroc × Landrace × Yorkshire, average body weight 7.86 ± 0.09 kg) at 28 days of age were divided into five groups and fed with a basal diet containing different levels of PRO (0, 30, 300, 600, and 1200 mg/kg) for 42 days. The diet (Table 7) was formulated based on National Research Council 2012 [52]. Pigs were raised alone in metabolic cages (1.05 m^2^) at appropriate temperature (26 ± 3 °C) and humidity (60 ± 5%) with ad-libitum access to water and foods, which were in a closed system. Before the formal experiment, there was a three-day adaptation period and the piglets had already been vaccinated against swine fever, blue ear, and pseudorabies at an appropriate time.

### 5.2. Dose Selection and Homogenization of PRO

Based on guidelines for evaluating the safety of livestock and poultry target animals to feed additives carried out by the ministry of Agriculture and Rural Affairs, the evaluation test should include at least three groups, including the control group, effective dose group, and multiple dose group (the multiple dose group is generally 10 times the effective dose). In certain production situations, the supplementation of additives may be increased, and the maximum dose for long-term toxicity testing is generally 30 to 50 times the optimal dose [53,54]. In order to provide a higher safe dose range for the product, we have chosen 48 times the optimal dose as the maximum dose. The homogenization of PRO is divided into two steps. Firstly, a certain amount of PRO was diluted by the carrier (extruded corn) to obtain the primary premix. Then, the primary premix is mixed with other raw ingredients powders to obtain a diet.

### 5.3. Sample Collection

Following defecation, fresh feces from each pig were collected into their individual valve bags, and next, 10 mL of diluted H_2_SO_4_ solution was injected into 0.1 kg of feces to fix excreta nitrogen during day 39–42 of the trial. On day 1, 22, and 43 in the morning, blood samples were taken from each pig by venipuncture, and then centrifuged at 3500× *g* at 4 °C (15 min) after 30 min. Then serums were obtained and stored at −20 °C for subsequent analysis. Then, pigs were slaughtered by electrical stunning to collect the remaining samples. After draining of the blood from the carotid artery, pigs were dissected to separate the organs and entire intestine. The small intestinal mucosal tissues were collected by scraping the mucosal layer with a glass slide and then stored at −80 °C [55]. Organs were taken from each pig for weighing and calculating the relative indices (liver, kidney, spleen, heart, and lungs) followed by removing any superficial fat or blood, and similar areas on each organ were collected to fix in paraformaldehyde for histology measurements (heart, liver, kidney, spleen, lungs, pancreas, thymus, mesenteric lymph nodes, stomach, duodenum, jejunum, ileum, and cecum).

### 5.4. Growth Performance and Diarrhea Rate Evaluation

The average daily feed intake (ADFI) was recorded daily and the body weight was recorded at the initial (IBW) and final (FBW) part of this study to calculate the feed/gain ratio (F:G) and average daily gain (ADG). Piglets with watery or sparse feces are considered as having diarrhea and the diarrhea rate is calculated by the previous formula [56].

### 5.5. Apparent Total Tract Nutrient Digestibility Analysis

The diet and fecal samples were used for the nutrient digestibility analysis after thawing, homogenizing, and freeze-drying, such as DM, CP, EE, and Ash [25]. The Cr_2_O_3_ was used as an external indicator, and all the procedures are according to the AOAC standard [57]. The GE was detected through the adiabatic calorimeter (LECO, St. Joseph, MI, USA). All contents were calculated by the following formula: 100 − 100 × (digesta nutrient × diet Cr_2_O_3_)/(diet nutrient × digesta Cr_2_O_3_) [58].

### 5.6. Hematology and Clinical Chemistry Analysis

Serum and whole blood were sent promptly to Ya‘an People’s Hospital (Yaan, China) for hematological parameters analysis. Hematology biochemical measured by automatic biochemical analyzer (7150, HITACHI, Tokyo, Japan) include aspartate aminotransferase (AST), alanine aminotransferase (ALT), blood glucose (GLU), blood urea nitrogen (BUN), creatinine (CREA), alkaline phosphatase (AKP), triglycerides (TG), total cholesterol (TC), albumin (ALB), globulin (GLO), total protein (TP), and total bilirubin (TBIL). Routine blood indexes included hemoglobin (HGB), white blood cell count (WBC), red blood cell count (RBC), platelet count (PLT), Hematocrit value (HCT), neutrophil count, and lymphocyte count and were measured by hematology analyzer (KX–21, SYSMEX, Wuxi, China).

### 5.7. Intestinal Morphologic Analysis

The small intestinal segments from the fixtive were dewaxed by graded anhydrous ethanol, dyed by hematoxylin and eosin (H&E), and sealed by a neutral resin size as described by Li [41]. Then, sections of the small intestinal samples were examined for crypt depth (CD), villus height (VH), and the ratio of VH to CD (V/C) by image processing and analysis system (Image-Pro Plus 6.0, Media Cybernetics, Rockville, MD, USA).

### 5.8. Histopathology Evaluations and Relative Weights of Organ

At terminal necropsy, organ weights (heart, lungs, spleen, liver, and kidney) were recorded and the relative weights of organs were calculated. Freshly harvested samples (heart, liver, kidney, spleen, lungs, pancreas, thymus, mesenteric lymph nodes, stomach, duodenum, jejunum, ileum, and cecum) on a standard selection were fixed in 4% paraformaldehyde and delivered to the Histopathology Laboratory (College of Veterinary Medicine, Southwest University of Science and Technology) where they were stained and made into sections as per the above methods of small intestinal sections [41,44]. Under a light microscope, the veterinary pathologist examined the sections for the determination of abnormalities. All relative weights of organs were calculated by following formula: A1/A2 [59]. A1: organ weight (g); A2: final live body weight (kg).

### 5.9. RNA Isolation, Reverse Transcription, and Real-Time Quantitative PCR

The RNA of small intestinal mucosal samples from each pig was extracted by Trizol (TAKARA, Gunma, Japan). Then, the RNA extracted from the intestinal mucosa was reverse transcribed into cDNA by PrimeScript RT Reagent Kit with gDNA Eraser (TaKaRa Biotechnology Co., Ltd., Dalian, China). PCR assays were conducted using Premix Ex TaqTM kits (Takara Biotechnology Co., Ltd., Dalian, China). The procedure of q-PCR is as follows: initial denaturation at 95 °C (30 s), followed by 40 cycles between 95 °C (5 s) and 60 °C (30 s). All procedures are based on previous methods [36]. The primers information is shown in Supplementary Material Table S1. The mRNA levels of important genes of pattern recognition receptor-related intestinal barrier genes, such as mucin 1, 2 (*MUC-1*, *MUC-2*), *Claudin-2*, and nutrient transporters, such as Oligopeptide Transporter 1 (*Petp-1*), fatty acid transport protein-1, 4 (*FATP-1*, *FATP-4)* were quantified by real-time PCR and the relative expression of each gene was calculated using the 2^−ΔΔCt^ method [60].

### 5.10. Statistical Analysis

The results were shown as the means and structural equation model (SEM). Data were subjected to one-way analysis of variance (ANOVA) followed by Tukey’s multiple-range test to determine significant differences among the groups at *p* < 0.05 with SPSS 27.0 (IBM, Chicago, IL, USA).

## Figures and Tables

**Table 1:** Effects of PRO on growth performance and diarrhea rate in weaned pigs.

Items	PRO (mg/kg)					SEM	*p* Value
	0	30	300	600	1200		
IBW (kg)	7.98	7.88	7.81	7.79	7.85	0.09	0.98
FBW (kg)	23.14	26.49	26.54	25.57	24.80	0.53	0.23
ADFI (g/d)	652.09	717.17	739.60	726.87	703.62	17.89	0.65
ADG (g/d)	364.53	443.07	445.88	432.41	403.55	11.85	0.17
F:G	1.79 ^a^	1.62 ^b^	1.66 ^b^	1.68 ^ab^	1.74 ^ab^	0.02	0.06
diarrhea rate%	11.90 ^a^	5.16 ^b^	4.76 ^b^	7.93 ^ab^	5.16 ^b^	0.01	0.09

^a, b^ mean values within a row with unlike superscript letters were significantly different (*p* < 0.05).

**Table 2:** Effects of PRO supplementation on apparent total tract digestibility of nutrients in weaned pigs.

Items	PRO (mg/kg)					SEM	*p* Value
	0	30	300	600	1200		
DM%	87.47 ^b^	89.34 ^a^	89.85 ^a^	89.16 ^a^	89.14 ^a^	0.002	0.01
EE%	73.00 ^c^	81.29 ^a^	81.31 ^a^	80.92 ^ab^	78.30 ^b^	0.007	0.01
GE%	88.15 ^b^	90.15 ^a^	90.66 ^a^	90.19 ^a^	89.85 ^a^	0.002	0.01
CP%	85.35	86.68	87.16	86.33	85.93	0.003	0.46
ASH%	53.84 ^d^	60.57 ^ac^	62.90 ^a^	57.18 ^b^	57.82 ^bc^	0.007	0.01

DM, dry matter; CP, crude protein; EE, ether extract; GE, gross energy. ^a, b, c^ mean values within a row with unlike superscript letters were significantly different (*p* < 0.05).

**Table 3:** Effects of PRO on intestinal morphology in weaned pigs.

Items	PRO (mg/kg)					SEM	*p* Value
	0	30	300	600	1200		
Duodenum							
VH (μm)	466.48 ^b^	601.93 ^a^	614.10 ^a^	582.13 ^a^	577.10 ^a^	12.49	0.01
CD (μm)	401.84 ^a^	266.28 ^b^	270.78 ^b^	271.15 ^b^	272.75 ^b^	11.57	0.01
V/C	1.25 ^b^	2.37 ^a^	2.41 ^a^	2.22 ^a^	2.25 ^a^	0.09	0.01
Jejunum							
VH (μm)	354.01 ^b^	458.77 ^a^	455.42 ^a^	416.73 ^a^	424.68 ^a^	11.44	0.02
CD (μm)	219.74	204.08	194.77	188.88	205.71	5.02	0.36
V/C	1.64 ^b^	2.36 ^a^	2.46 ^a^	2.32 ^a^	2.15 ^a^	0.07	0.01
Ileum							
VH (μm)	305.30	322.80	340.48	292.03	302.50	7.16	0.22
CD (μm)	153.81	145.43	164.06	145.68	128.62	4.27	0.10
V/C	2.13	2.38	2.18	2.22	2.48	0.05	0.17

^a, b^ mean values within a row with unlike superscript letters were significantly different (*p* < 0.05).

**Table 4:** Effects of PRO on Clinical chemistry parameters in weaned pigs.

Items	PRO (mg/kg)					SEM	*p* Value
	0	30	300	600	1200		
Day 1							
Total protein (g/L)	46.95	47.92	47.37	45.35	45.72	0.49	0.43
Albumin (g/L)	36.83	38.33	39.62	35.63	37.45	0.50	0.12
Globulin (g/L)	10.12	9.58	7.75	9.72	8.27	0.40	0.28
TBILI (μmol/L)	3.10	1.35	1.15	2.23	2.02	0.31	0.32
ALT (U/L)	33.80	31.87	25.12	25.50	29.28	1.72	0.43
AST (U/L)	21.78	23.87	18.12	18.72	16.76	1.15	0.29
AKP (U/L)	342.17	339.85	349.33	328.33	353.08	12.26	0.98
BUN (mmol/L)	2.75	2.94	2.67	2.91	3.17	0.17	0.92
Creatinine (μmol/L)	106	118	108.83	114	116	2.37	0.49
Glucose (mmol/L)	6.97	6.73	7.41	6.87	6.81	0.15	0.63
TC (mmol/mL)	2.03	1.89	2.08	1.97	1.94	0.06	0.91
Day 22							
Total protein (g/L)	39.55	39.80	41.18	40.08	39.28	0.44	0.73
Albumin (g/L)	27.53	28.43	29.05	27.85	27.23	0.42	0.69
Globulin (g/L)	12.02	11.37	12.13	12.23	12.05	0.30	0.91
TBILI (μmol/L)	0.75	0.63	0.55	0.73	0.70	0.05	0.78
ALT (U/L)	49.13	60.17	59.58	50.05	49.23	2.65	0.47
AST (U/L)	38.75	45.83	49.62	45.93	39.05	2.26	0.63
AKP (U/L)	189.23	225.82	211.70	189.82	219.33	10.00	0.72
BUN (mmol/L)	3.78	2.70	3.16	3.42	3.76	0.19	0.36
Creatinine (μmol/L)	82.50	83.17	83.83	94.50	87.17	1.93	0.26
Glucose (mmol/L)	5.94	5.91	6.14	6.61	6.47	0.13	0.33
TC (mmol/mL)	1.80 ^a^	1.49 ^b^	1.65 ^ab^	1.72 ^ab^	1.59 ^ab^	0.04	0.08
Day 43							
Total protein (g/L)	49.03	46.33	49.43	48.08	45.45	0.60	0.16
Albumin (g/L)	38.42	38.77	40.02	39.50	37.65	0.68	0.86
Globulin (g/L)	10.62	9.23	9.42	8.58	8.58	0.33	0.28
TBILI (μmol/L)	0.90	1.12	0.82	0.92	0.57	0.08	0.30
ALT (U/L)	53.65 ^ab^	64.10 ^a^	64.62 ^a^	44.40 ^b^	45.25 ^b^	2.72	0.02
AST (U/L)	46.53	65.95	67.96	60.80	34.60	5.35	0.23
AKP (U/L)	175.55	187.42	184.60	169.73	190.72	6.48	0.86
BUN (mmol/L)	2.60	2.74	2.37	2.18	1.93	0.14	0.42
Creatinine (μmol/L)	98.83	107.50	100.00	102.50	99.67	2.29	0.78
Glucose (mmol/L)	7.86	7.78	6.98	8.16	7.07	0.29	0.68
TC (mmol/mL)	2.33 ^a^	1.93 ^b^	2.34 ^b^	2.37 ^b^	2.09 ^ab^	0.06	0.08

AKP: Alkaline phosphatase; BUN: Blood urea nitrogen; ALT: Alanine aminotransferase. AST: Aspartate aminotransferase. TC: Total cholesterol. ^a, b^ mean values within a row with unlike superscript letters were significantly different (*p* < 0.05).

**Table 5:** Effects of PRO on hematological parameters in weaned pigs.

Items	PRO (mg/kg)					SEM	*p* Value
	0	30	300	600	1200		
Day 1							
WBC (10^9^·L^−1^)	11.16	10.81	12.10	12.05	10.17	0.57	0.82
RBC (10^12^·L^−1^)	7.07	7.19	6.99	6.81	7.10	0.09	0.79
HGB (g·L^−1^)	121.50 ^b^	140.00 ^a^	130.80 ^ab^	131.00 ^ab^	131.33 ^ab^	1.94	0.03
HCT (%)	39.58 ^b^	45.38 ^a^	42.72 ^ab^	42.16 ^ab^	42.73 ^ab^	0.58	0.02
PLT (10^3^·µL^−1^)	627.33	465.00	415.50	513.33	475.17	40.74	0.58
Lymphocytes (10^9^·L^−1^)	7.00	6.54	7.10	6.48	6.53	0.39	0.98
Neutrophils (10^9^·L^−1^)	3.63	3.61	4.30	3.50	2.09	0.42	0.58
Day 22							
WBC (10^9^·L^−1^)	22.38	19.20	19.60	19.50	21.17	1.21	0.92
RBC (10^12^·L^−1^)	6.48	6.41	6.20	6.42	6.33	0.10	0.92
HGB (g·L^−1^)	109.00	115.00	107.50	111.20	110.83	1.85	0.78
HCT (%)	34.97	37.00	34.87	36.24	36.28	0.57	0.74
PLT (10^3^·µL^−1^)	579.33	421.50	379.48	464.40	498.50	26.45	0.13
Lymphocytes (10^9^·L^−1^)	4.18	7.68	3.73	8.41	3.88	1.12	0.55
Neutrophils (10^9^·L^−1^)	9.80	6.56	7.63	8.12	7.32	0.70	0.68
Day 43							
WBC (10^9^·L^−1^)	21.10	15.32	16.46	19.68	17.40	0.94	0.11
RBC (10^12^·L^−1^)	7.47	7.12	6.91	7.01	7.10	0.09	0.36
HGB (g·L^−1^)	128.50	122.83	117.17	121.33	120.67	1.94	0.48
HCT (%)	47.75	44.78	42.78	45.50	44.38	0.86	0.49
PLT (10^3^·µL^−1^)	463.33	369.00	442.50	483.33	435.00	19.24	0.42
Lymphocytes (10^9^·L^−1^)	13.85	10.25	11.44	12.16	10.62	0.61	0.44
Neutrophils (10^9^·L^−1^)	2.73	4.15	3.91	3.42	2.82	0.36	0.68

WBC: White blood count; RBC: Red blood count; HGB: hemoglobin; PLT: Platelet count; HCT: Hematocrit value. ^a, b^ mean values within a row with unlike superscript letters were significantly different (*p* < 0.05).

**Table 6:** Effects of PRO on relative weights of the organ in weaned pigs.

Items	PRO (mg/kg)					SEM	*p* Value
	0	30	300	600	1200		
Heart, g/kg	4.93	5.11	5.13	5.16	5.08	0.09	0.95
Liver, g/kg	24.83	23.09	23.68	25.00	23.13	0.42	0.45
Spleen, g/kg	1.47	1.40	1.68	1.71	1.59	0.07	0.60
Lung, g/kg	11.63	11.19	11.91	12.12	11.77	0.38	0.96
Kidney, g/kg	4.87	4.53	4.83	4.76	5.04	0.08	0.39

**Table 7:** Ingredients and nutrient composition of the basal diet.

Ingredients	%	Nutrient Level	Contents
Corn	31.06	Digestible energy (calculated, MJ/kg)	3.50
Extruded corn	30.54	Crude Protein (%)	18.76
Soybean meal	8.00	Calcium (%)	0.76
Extruded full-fat soybean	9.95	Available phosphorus (%)	0.37
Fish meal	5.00	Lysine	1.30
Whey powder	5.00	Methionine	0.37
Soybean protein concentrate	4.80	Methionine + cysteine	0.66
Soybean oil	1.29	Threonine	0.76
Sucrose	2.00	Tryptophan	0.22
Limestone	0.64		
Dicalcium phosphate	0.34		
NaCl	0.20		
L-Lysine HCl (78%)	0.30		
DL-Methionine	0.06		
L-Threonine (98.5%)	0.01		
Tryptophan (98%)	0.01		
Chloride choline	0.15		
Vitamin premix ^1^	0.05		
Mineral premix ^2^	0.30		
Total	100		

Based on dry matter. ^1^ The vitamin premix contain: VA, VD3, VE, VK3, VB2, and VB12; ^2^ The mineral premix contain: Mn, Cu, I, Zn, Fe, Se, and Ca.

## Data Availability

The data presented in this study are available on request from the corresponding author.

## Supplementary Materials

The following supporting information can be downloaded at: https://www.mdpi.com/article/10.3390/toxins15090558/s1, Table S1: Primers sequences used for quantitative RT-PCR.

## References

[1] Rauf A., Imran M., Abu-Izneid T., Haq I.U., Patel S., Pan X., Naz S., Silva A.S., Saeed F., Suleria H.A.R. (2019). Proanthocyanidins: A comprehensive review. Biomed. Pharmacother..

[2] Rue E.A., Rush M.D., van Breemen R.B. (2018). Procyanidins: A comprehensive review encompassing structure elucidation via mass spectrometry. Phytochem. Rev..

[3] Qi Q., Chu M., Yu X., Xie Y., Li Y., Du Y., Liu X., Zhang Z., Shi J., Yan N. (2023). Anthocyanins and Proanthocyanidins: Chemical Structures, Food Sources, Bioactivities, and Product Development. Food Rev. Int..

[4] Liu X., Renard C.M.G.C., Rollan-Sabaté A., Le Bourvellec C. (2021). Exploring interactions between pectins and procyanidins: Structure-function relationships. Food Hydrocoll..

[5] Saucier C., Mirabel M., Daviaud F., Longieras A., Glories Y. (2001). Rapid fractionation of grape seed proanthocyanidins. J. Agric. Food Chem..

[6] Bagchi D., Sen C.K., Ray S.D., Das D.K., Bagchi M., Preuss H.G., Vinson J.A. (2003). Molecular mechanisms of cardioprotection by a novel grape seed proanthocyanidin extract. Mutat. Res..

[7] Han X., Guo J.L., Yin M.W., Liu Y., You Y., Zhan J., Huang W. (2020). Grape extract activates brown adipose tissue through pathway involving the regulation of gut microbiota and bile acid. Mol. Nutr. Food Res..

[8] Xia E.Q., Deng G.F., Guo Y.J., Li H.B. (2010). Biological activities of polyphenols from grapes. Int. J. Mol. Sci..

[9] Irak K., Yildirim S., Mert H., Mert N. (2018). Grape seed extract effects on serum amylase levels and immunohistochemical alterations in streptozotocin-Induced diabetic rats. Cell Mol. Biol..

[10] Wang Y.H., Ge B., Yang X.L., Zhai J., Yang L.N., Wang X.X., Liu X., Shi J.C., Wu Y.J. (2011). Proanthocyanidins from grape seeds modulates the nuclear factor-kappa B signal transduction pathways in rats with TNBS-induced recurrent ulcerative colitis. Int. Immunopharmacol..

[11] Pascual-Serrano A., Arola-Arnal A., Suárez-García S., Bravo F.I., Suárez M., Arola L., Bladé C. (2017). Grape seed proanthocyanidin supplementation reduces adipocyte size and increases adipocyte number in obese rats. Int. J. Obes..

[12] Pajuelo D., Díaz S., Quesada H., Fernández-Iglesias A., Mulero M., Arola-Arnal A., Salvadó M.J., Bladé C., Arola L. (2011). Acute administration of grape seed proanthocyanidin extract modulates energetic metabolism in skeletal muscle and BAT mitochondria. J. Agric. Food Chem..

[13] Chen T., Shen M., Yu Q., Chen Y., Wen H., Lu H., Chen S., Xie J. (2022). Purple red rice anthocyanins alleviate intestinal damage in cyclophosphamide-induced mice associated with modulation of intestinal barrier function and gut microbiota. Food Chem..

[14] Casanova-Martí À., González-Abuín N., Serrano J., Blay M.T., Terra X., Frost G., Pinent M., Ardévol A. (2020). Long Term Exposure to a Grape Seed Proanthocyanidin Extract Enhances L-Cell Differentiation in Intestinal Organoids. Mol. Nutr. Food Res..

[15] Bode A.M., Dong Z. (2015). Toxic phytochemicals and their potential risks for human cancer. Cancer Prev. Res..

[16] Na Y., Yang F.R. (2002). Overview of research on development and utilization of medicinal plant resources in foreign countries. For. Investig. Des..

[17] Guldiken B., Ozkan G., Catalkaya G., Ceylan F.D., Yalcinkaya I.E., Capanoglu E. (2018). Phytochemicals of herbs and spices: Health versus toxicological effects. Food Chem. Toxicol..

[18] Song P., Zhang R., Wang X., He P., Tan L., Ma X. (2011). Dietary grape-seed procyanidins decreased postweaning diarrhea by modulating intestinal permeability and suppressing oxidative stress in rats. J. Agric. Food Chem..

[19] Wu Y., Ma N., Song P., Ting H., Crystal L., Yueyu B., Aizhong Z., Xi M. (2019). Grape Seed Proanthocyanidin Affects Lipid Metabolism via Changing Gut Microflora and Enhancing Propionate Production in Weaned Pigs. J. Nutr..

[20] Soliz-Rueda J.R., López-Fernández-Sobrino R., Bravo F.I., Aragonès G., Suarez M., Muguerza B. (2022). Grape Seed Proanthocyanidins Mitigate the Disturbances Caused by an Abrupt Photoperiod Change in Healthy and Obese Rats. Nutrients.

[21] Mas-Capdevila A., Iglesias-Carres L., Arola-Arnal A., Suárez M., Bravo F.I., Muguerza B. (2020). Changes in arterial blood pressure caused by long-term administration of grape seed proanthocyanidins in rats with established hypertension. Food Funct..

[22] Yang J.Y., Zhang H.J., Wang J., Wu S.G., Yue H.Y., Jiang X.R., Qi G.H. (2017). Effects of dietary grape proanthocyanidins on the growth performance, jejunum morphology and plasma biochemical indices of broiler chicks. Animal.

[23] Silvan J.M., Mingo E., Hidalgo M., de Pascual-Teresa S., Carrascosa A.V., Martinez-Rodriguez A.J. (2013). Antibacterial activity of a grape seed extract and its fractions against *Campylobacter* spp.. Food Control..

[24] Cosansu S., Juneja V.K., Osoria M., Mukhopadhyay S. (2019). Effect of grape seed extract on heat resistance of Clostridium perfringens vegetative cells in sous vide processed ground beef. Food Res..

[25] Gessner D.K., Bonarius M., Most E., Fiesel A., Eder K. (2017). Effects of polyphenol-rich plant products from grape or hop as feed supplements on the expression of inflammatory, antioxidative, cytoprotective and endoplasmic reticulum stress-related genes and the antioxidative status in the liver of piglets. J. Anim. Physiol. Anim. Nutr..

[26] Yang K.M., Jiang Z.Y., Zheng C.T., Wang L., Yang X.F. (2014). Effect of Lactobacillus plantarum on diarrhea and intestinal barrier function of young piglets challenged with enterotoxigenic *Escherichia coli* K88. J. Anim. Sci..

[27] McLamb B.L., Gibson A.J., Overman E.L., Stahl C., Moeser A.J. (2013). Early weaning stress in pigs impairs innate mucosal immune responses to enterotoxigenic *E. coli* challenge and exacerbates intestinal injury and clinical disease. PLoS ONE.

[28] Pluske J.R., Thompson M.J., Atwood C.S., Bird P.H., Williams I.H., Hartmann P.E. (1996). Maintenance of villus height and crypt depth, and enhancement of disaccharide digestion and monosaccharide absorption, in piglets fed on cow’s whole milk after weaning. Br. J. Nutr..

[29] Pluske J.R., Williams I.H., Aherne F.X. (1996). Maintenance of villous height and crypt depth in piglets by providing continuous nutrition after weaning. Anim. Sci..

[30] Xin J.L., In H.K. (2018). Low dose of coated zinc oxide is as effective as pharmacological zinc oxide in promoting growth performance, reducing fecal scores, and improving nutrient digestibility and intestinal morphology in weaned pigs. Anim. Feed. Sci. Technol..

[31] Viveros A., Chamorro S., Pizarro M., Arija I., Centeno C., Brenes A. (2011). Effects of dietary polyphenol-rich grape products on intestinal microflora and gut morphology in broiler chicks. Poult. Sci..

[32] Sehm J., Lindermayer H., Dummer C., Treutter D., Pfaffl M.W. (2007). The influence of polyphenol rich apple pomace or red-wine pomace diet on the gut morphology in weaning piglets. J. Anim. Physiol. Anim. Nutr..

[33] Xu X., Wei Y., Hua H., Jing X., Zhu H., Xiao K., Zhao J., Liu Y. (2022). Polyphenols Sourced from Ilex latifolia Thunb. Relieve Intestinal Injury via Modulating Ferroptosis in Weanling Piglets under Oxidative Stress. Antioxidants.

[34] Garrett W.S., Gordon J.I., Glimcher L.H. (2010). Homeostasis and inflammation in the intestine. Cell.

[35] Yamamoto-Furusho J.K., Ascaño-Gutiérrez I., Furuzawa-Carballeda J., Fonseca-Camarillo G. (2015). Differential Expression of MUC12, MUC16, and MUC20 in Patients with Active and Remission Ulcerative Colitis. Mediat. Inflamm..

[36] Liu Y., Yu X., Zhao J., Zhang H., Zhai Q., Chen W. (2020). The role of MUC2 mucin in intestinal homeostasis and the impact of dietary components on MUC2 expression. Int. J. Biol. Macromol..

[37] Arike L., Hansson G.C. (2016). The Densely O-Glycosylated MUC2 Mucin Protects the Intestine and Provides Food for the Commensal Bacteria. J. Mol. Biol..

[38] Mitchell R.W., Hatch G.M. (2009). Regulation of cardiolipin biosynthesis by fatty acid transport protein-1 IN HEK 293 cells. Biochim. Biophys. Acta..

[39] Herrmann T., van der Hoeven F., Grone H.J., Stewart A.F., Langbein L., Kaiser I., Liebisch G., Gosch I., Buchkremer F., Drobnik W. (2003). Mice with targeted disruption of the fatty acid transport protein 4 (Fatp 4, Slc27a4) gene show features of lethal restrictive dermopathy. J. Cell Biol..

[40] Mooij M.G., de Koning B.E., Lindenbergh-Kortleve D.J., Simons-Oosterhuis Y., van Groen B.D., Tibboel D., Samsom J.N., de Wildt S.N. (2016). Human Intestinal PEPT1 Transporter Expression and Localization in Preterm and Term Infants. Drug Metab. Dispos..

[41] Li Y.P., Jiang X.R., Wei Z.X., Cai L., Yin J.D., Li X.L. (2020). Effects of soybean isoflavones on the growth performance, intestinal morphology and antioxidative properties in pigs. Animal.

[42] Danicke S., Doll S.A. (2010). probiotic feed additive containing spores of Bacillus subtilis and B. licheniformis does not prevent absorption and toxic effects of the Fusarium toxin deoxynivalenol in piglets. Food Chem. Toxicol..

[43] Tyburczy C., Kothapalli K.S.D., Park W.J. (2010). Negligible effect of dietary arachidonic acid (ARA) levels on growth, clinical chemistry and immune function in domestic piglets. Brit. J. Nutr..

[44] Herfel T.M., Jacobi S.K., Lin X., Walker D.C., Jouni Z.E., Odle J. (2009). Safety evaluation of polydextrose in infant formula using a suckling piglet model. Food Chem. Toxicol..

[45] Chen Y., Chen T., Luo Y., Fan J., Zhang M., Zhao Q., Nan Y., Liu B., Zhou E.M. (2020). Synthetic Peptides Containing Three Neutralizing Epitopes of Genotype 4 Swine Hepatitis E Virus ORF2 induced Protection against Swine HEV Infection in Rabbit. Vaccines.

[46] Dubreuil P., Lapierre H. (1997). Biochemistry reference values for Quebec lactating dairy cows, nursing sows, growing pigs and calves. Can. J. Vet. Res..

[47] Cooper C.A., Moraes L.E., Murray J.D., Owens S.D. (2014). Hematologic and biochemical reference intervals for specific pathogen free 6-week-old Hampshire-Yorkshire crossbred pigs. J. Anim. Sci. Biotechnol..

[48] Zhang Q., Li J., Huang T., Zhang Y., Xu W., Huang L., Ai H., Yang B. (2020). Impacts of Mycoplasma loads and lung lesions on immune and hematological statuses of pigs in an eight-breed cross heterogeneous population. J. Anim. Sci..

[49] Quesada H., del Bas J.M., Pajuelo D., Díaz S., Fernandez-Larrea J., Pinent M., Arola L., Salvadó M.J., Bladé C. (2009). Grape seed proanthocyanidins correct dyslipidemia associated with a high-fat diet in rats and repress genes controlling lipogenesis and VLDL assembling in liver. Int. J. Obes..

[50] Wang J., Sun F., Tang S., Zhang S., Lv P., Li J., Cao X. (2017). Safety assessment of vitacoxib: Acute and 90-day sub-chronic oral toxicity studies. Regul. Toxicol. Pharmacol..

[51] Mirza A.C., Panchal S.S. (2019). Safety evaluation of syringic acid: Subacute oral toxicity studies in Wistar rats. Heliyon.

[52] National Research Council (U.S.) (2012). Nutrient Requirements of Swine.

[53] Wei Z., Yu B., Huang Z., Luo Y., Zheng P., Mao X., Yu J., Luo J., Yan H., He J. (2023). Effect of 3-caffeoylquinic acid on growth performance, nutrient digestibility, and intestinal functions in weaned pigs. J. Anim. Sci..

[54] Chen J., Li Y., Yu B., Chen D., Mao X., Zheng P., Luo J., He J. (2018). Dietary chlorogenic acid improves growth performance of weaned pigs through maintaining antioxidant capacity and intestinal digestion and absorption function. J. Anim. Sci..

[55] Christensen B., Huber L.A. (2022). The effects of creep feed composition and form and nursery diet complexity on small intestinal morphology and jejunal mucosa-specific enzyme activities after weaning in pigs. J. Anim. Sci..

[56] Zhou Y., Luo Y., Yu B., Zheng P., Yu J., Huang Z., Mao X., Luo J., Yan H., He J. (2022). Agrobacterium sp. ZX09 β-Glucan Attenuates Enterotoxigenic *Escherichia coli*-Induced Disruption of Intestinal Epithelium in Weaned Pigs. Int. J. Mol. Sci..

[57] Latimer G.W. (2016). Official Methods of Analysis of AOAC International.

[58] Lu H., Shin S., Kuehn I., Bedford M., Rodehutscord M., Adeola O., Ajuwon K.M. (2020). Effect of phytase on nutrient digestibility and expression of intestinal tight junction and nutrient transporter genes in pigs. J. Anim. Sci..

[59] Ji F., Yang H., Wang Q., Li J., Zhou H., Liu S. (2023). Porcine intestinal antimicrobial peptide as an in-feed antibiotic alternative improves intestinal digestion and immunity by shaping the gut microbiota in weaned piglets. Anim. Nutr..

[60] Wan J., Zhang J., Chen D., Yu B., Mao X., Zheng P., Yu J., Luo J., He J. (2018). Alginate oligosaccharide-induced intestinal morphology, barrier function and epithelium apoptosis modifications have beneficial effects on the growth performance of weaned pigs. J. Anim. Sci. Biotechnol..

