# Supplementary material for: Functional Characterization and Toxicological Study of Proanthocyanidins in Weaned Pigs
Source: Toxins (Basel). 2023 Sep 7;15(9):558. doi: 10.3390/toxins15090558 (PMC10535313; doi:10.3390/toxins15090558)
Supplement: Supplementary file 1 [file toxins-15-00558-s001.zip › toxins-2570474-SI.pdf]

Supplementary Materials

Functional Characterization and Toxicological Study of Proanthocyanidins in Weaned Pigs

Jiahao Liu, Yong Qiao, Bing Yu, Yuheng Luo, Zhiqing Huang, Xiangbing Mao, Jie Yu, Ping Zheng, Hui Yan, Yan Li, and Jun He

Table S1. Primers sequences used for quantitative RT-PCR.

Gene	Primer sequence (5′–3′)
β-Actin	F: TGGAACGGTGAAGGTGACAGC<br>R: GCTTTTGGGAAGGCAGGGACT
FATP-1	F:GGAGTAGAGGGCAAAGCAGG<br>R:AGGTCTGGCGTGGGTCAAAG
FATP-4	F: TTCATCAAGACGGTCAGGCG<br>R: AGACGGTGGCAGCGAATAAG
MUC1	F:GGTGCTGGTCTGTATTCTGGT<br>R:TGCCCACAGTTCTTTCGTCG
MUC2	F:GGACGACACCATCTACCTCAC<br>R:CCAGCTCGGGAATAGACCTT
PepT1-F	F:CAGACTTCGACCACAACGGA<br>R:TTATCCCGCCAGTACCCAGA
Claudin-2	F:CTCCCTGTTCTCCCTGATAGC<br>R:TCACTCTTGGCTTTGGGTGG

FATP-1, 4, fatty acid transport protein-1,4; PepT1, Oligopeptide Transporter 1; MUC1,2, mucin 1,2.
